# Moyamoya: An Update and Review

**DOI:** 10.7759/cureus.10994

**Published:** 2020-10-16

**Authors:** James A Berry, Vladimir Cortez, Harjyot Toor, Harneel Saini, Javed Siddiqi

**Affiliations:** 1 Neurosurgery, Riverside University Health System Medical Center, Moreno Valley, USA; 2 Neurosurgery, Desert Regional Medical Center, Palm Springs, USA; 3 Osteopathic Medicine, Lake Erie College of Osteopathic Medicine, Erie, USA; 4 Neurosurgery, Arrowhead Regional Medical Center, Colton, USA; 5 Neurosurgery, California University of Science and Medicine, Colton, USA

**Keywords:** moyamoya, cerebro-vascular surgery

## Abstract

This article is a clinical review of Moyamoya disease (MMD) and Moyamoya syndrome (MMS). We review the incidence, epidemiology, pathology, historical context, clinical and radiographic findings, diagnostic imaging modalities, radiographic grading systems, the effectiveness of medical, interventional, and surgical treatment, and some of the nuances of surgical treatment options. This article will help pediatricians, neurologists, neurosurgeons, and other clinical practitioners who are involved in caring for patients with this rare clinical entity.

MMD is an intrinsic primary disease process that causes bilateral progressive stenosis of the anterior intracranial circulation with the involvement of the proximal portions of the intracranial internal carotid artery (ICA) extending to involve the proximal portions of the anterior cerebral artery (ACA) and middle cerebral artery (MCA); posterior circulation involvement is very rare. This causes a compensatory response where large numbers of smaller vessels such as the lenticulostriate arteries begin to enlarge and proliferate, which gives the angiographic appearance of a "Puff of Smoke", which is translated into Japanese as "Moyamoya”. MMS is a secondary process that occurs in response to another underlying pathological process that causes stenosis of intracranial blood vessels, such as radiation. For example, an external source of radiation causes stenosis of the ICA with a compensatory response of smaller blood vessels, which then enlarge and proliferate in response and has the same "Puff of Smoke" appearance on the diagnostic cerebral angiogram (DCA).

Histological findings include an irregular internal elastic lamina with luminal narrowing, hyperplasia of the tunica media, and intimal thickening with vacuolar degeneration in smooth muscle cells in the tunica media. Compensation for diminishing blood supply occurs through angiogenesis, which causes the proliferation and enlargement of smaller collateral blood vessels to increase blood supply to under-perfused areas of the brain.

MMD is rare in the United States, with just 0.086 newly diagnosed cases per 100,000 individuals per year, which is approximately one per million new cases annually. Risk factors for MMD include Eastern Asian ancestry and predisposing conditions such as neurofibromatosis and Down's syndrome.

Clinically, patients often present with stroke signs and symptoms from cerebral ischemia. The proliferation of collateral blood vessels within the basal ganglia can produce movement disorders. Catheter-based DCA is the current gold standard for obtaining a diagnosis. CT perfusion allows preoperative identification of ischemic vascular territories, which may be amenable to surgical intervention. MRI enables rapid detection of acute ischemic stroke using diffusion-weighted Imaging (DWI) and apparent diffusion coefficient (ADC) sequences to assess for any diffusion restriction. Non-contrast CT of the head is used to rule out acute hemorrhage in the presentation of a progressive neurological deficit.

The treatment option for Moyamoya is generally surgical; medical treatment has failed to halt disease progression and neuro-interventional techniques such as attempted stenting of stenosed vessels have failed. Surgical options include direct and indirect cerebrovascular bypass.

## Introduction and background

Moyamoya disease (MMD) is a chronic cerebrovascular disorder characterized by a progressive narrowing of the intracranial portions of the distal internal carotid artery (ICA) and the initial proximal components of the middle cerebral artery (MCA) and anterior cerebral artery (ACA). The involvement of the vertebrobasilar posterior circulation is very rare [1].

MMD is a primary intrinsic pathological disease process with an incompletely understood etiology. The progressive stenosis causes decreased cerebral blood flow to the brain parenchyma, causing ischemic and a compensatory vascular response. The cerebral vasculature attempts to compensate by developing collateral circulation, and smaller blood vessels begin to enlarge, and hypertrophy becomes more visible on the diagnostic cerebral angiogram (DCA). The formation of collateral blood vessels gives the angiographic appearance of a “puff of smoke”, which is roughly translated as “Moyamoya” in Japanese. This is the identifying angiographic characteristic of the disorder. It was previously believed that these collateral vessels represented the creation of entirely new vessels; however, recent studies suggest it is the enlargement of preexisting micro-perforator arteries such as the lenticulostriate and thalamoperforating arteries.

MMD almost exclusively affects the anterior circulation, beginning in the distal portions of the intracranial ICA and the proximal portions of the MCA and ACA. As the disease progresses, hypo-perfused brain parenchyma begins to recruit vessels from the leptomeninges and eventually form anastomotic branches of the external carotid artery (ECA).

The arterial angiographic findings of progressive stenosis in the anterior circulation by definition occurs bilaterally in MMD, and unilateral findings are classified as Moyamoya syndrome (MMS). MMS is a secondary process that occurs in response to another pathological process. An example that illustrates this is patients who receive radiation to their head and neck for a malignancy going on to develop significant radiation-induced stenosis of their ICA. As a result, these patients develop MMS, where the body attempts to compensate for the ICA stenosis by the hypertrophy of existing smaller blood vessels, such as the lenticulostriate perforating arteries. Typically, MMS has unilateral involvement, which differentiates it from MMD.

## Review

Pathology

**Figure 1:**
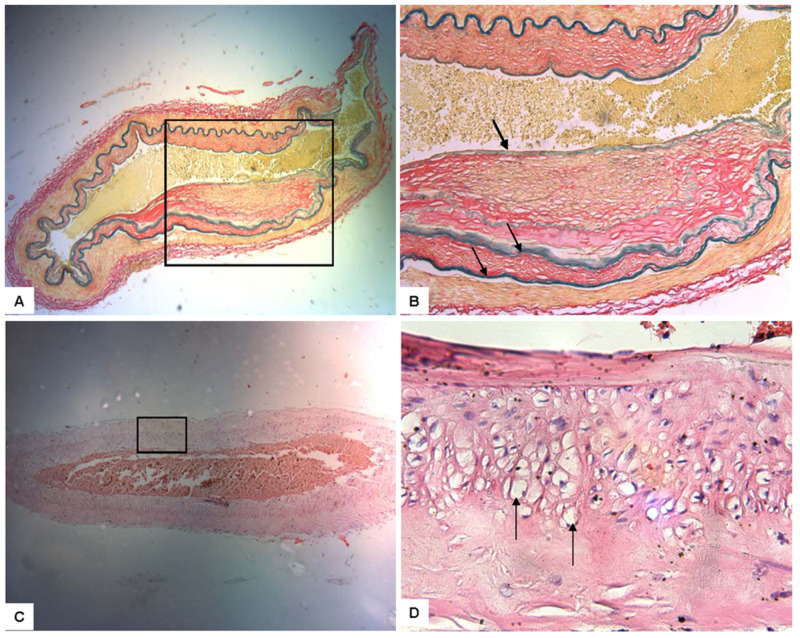
Cross-section of a Moyamoya-affected middle cerebral artery post-mortem specimen Black arrows indicate the irregular internal elastic lamina. A and B: the lumen is narrowed, there is hyperplasia of the tunica media and intimal thickening. C and D: vacuolar degeneration in smooth muscle cells in the tunica media Images used with permission of Emma Darkin, Staff EO

**Figure 2:**
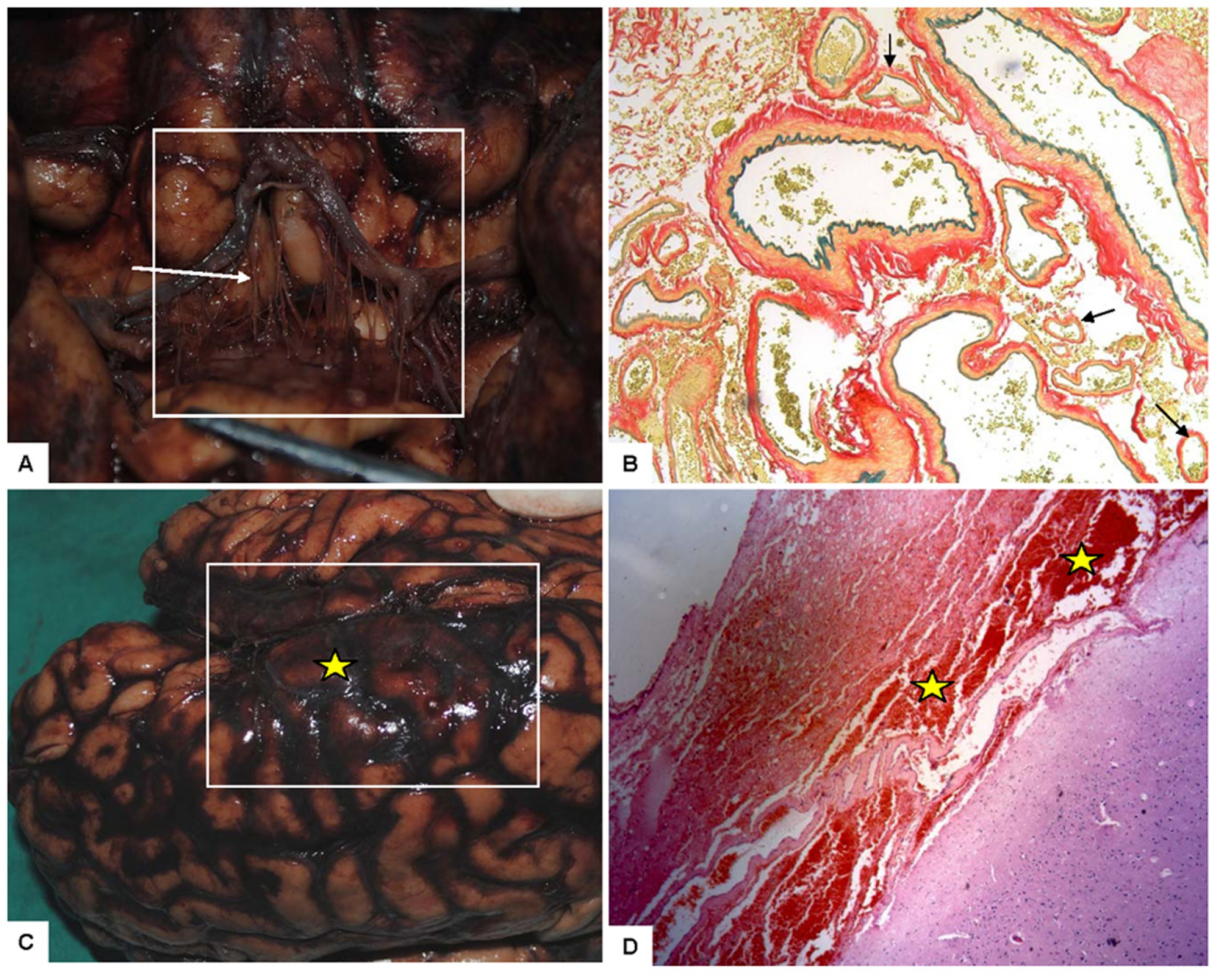
Gross and microscopic features of Moyamoya pathology A: an array of enlarged and dilated collateral Moyamoya vessels from a pathological post-mortem specimen. B: arrows demonstrate enlarging Moyamoya collateral vessels: C: subarachnoid hemorrhage presents next to collateral vessels. D: the presence of subarachnoid hemorrhage in the cross-section Images used with permission of Emma Darkin, Staff EO

The exact pathophysiologic mechanism leading to the development of MMD is not completely understood; however, several studies, which we cite throughout this paragraph, have discovered factors that may increase the risk of developing this unique pathology. The stenosis in MMD is caused by an alternative process, rather than the more commonly encountered atherosclerotic and inflammatory causes [2]. Specimens instead show hyperplasia of proliferating smooth muscle cells, particularly in the tunica media of the arterial wall [3]. The tunica media appear to be impaired by an irregular layering of elastic lamina [2]. Further histological specimens demonstrate micro-aneurysm formation, providing an explanation for the higher incidence of hemorrhage in MMD patients, particularly among adults [4]. The evidence suggests that apoptosis, not necrosis, is responsible for the detrimental changes in the arterial wall [5]. Findings show that the following cascade is mediated by a caspase mechanism, and an underlying autoimmune process may be responsible for the disease [6]. Elevated autoantibodies have been discovered against APP, GPS1, STRA13, CTNB1, ROR1, and EDIL3, leading to the aforementioned apoptotic process [7]. The histopathological changes associated with MMD are shown in Figure 1. Serum studies in patients with the active disease show higher circulating plasma concentrations of vascular angiogenesis, producing growth factors such as vascular endothelial growth factor (VEGF), matrix metalloproteinase (MMP), hepatocyte growth factor, and interleukin-1B [8]. The enlargement and dilation of these pre-existing small perforator branches are fragile and prone to aneurysmal formation [9]. As vessel perforators progressively dilate, the wall experiences increased stress leading to the fragmentation of the elastic layer; as the wall stretches, it becomes weaker and has the potential for aneurysmal dissection, which can occlude the vessel lumen and potentially rupture causing hemorrhage, as seen in Figure 2 [4].

The dysfunction of endothelial colony-forming cells (ECFC), also known as endothelial progenitor cells, is believed to play an important role in the development of MMD. The mitochondria of ECFCs in patients with MMD were discovered to have a significant structural and functional impairment, resulting in decreased oxygen consumption rates [10]. Enlarging collateral vessels, in particular lenticulostriate vessels, that pierce and perfuse the basal ganglia, have been associated with the development of complex movement disorders. Hemiballismus-type movements with subthalamic nucleus involvement have been documented [11]. It is unclear if the development of these complex movement disorders is due to enlarging vessels disrupting connections between fibers and nuclei or if it is caused by micro-ischemic changes from diminishing perfusion due to progressive stenosis. Cross-sectional histologic assessment of arteries from affected patients demonstrates an atypically thickened tunica intima and a decreased tunica media layer [12].

A recent large scale international meta-analysis performed across East Asia identified a significant genetic risk factor for the development of MMD in China, Japan, and South Korea. The annual incidence of newly diagnosed cases in these regions can be as high as 6.03 per 100,000 [13,14]. Patients with MMD have an altered pattern of expression of circular RNA in the mitogen-activated protein kinase signaling pathway [15]. Polymorphisms in ring finger protein 213 (RNF213) appear to make patients in certain populations in East Asia more susceptible to developing MMD [16]. Aside from vessel anomalies, analysis of cerebrospinal fluid (CSF) from MMD patients showed that the proliferation of new collateral vessels was linked to the downregulation of apolipoprotein-E and J [17]. These findings suggest that lipid metabolism may be either a reaction to or a contributory factor in the development and progression of MMD.

Clinical features

Ischemic events are the most common initial presentation and are more prevalent in the pediatric population [18]. Hemorrhagic presentation, particularly clinically significant intracerebral hemorrhage, is rare in the pediatric population. Phase-sensitive MRI has detected a high incidence of clinically silent asymptomatic micro-bleeds in pediatric MMD [19]. It is less common that patients will develop hemorrhagic events; however, if they do, it is more likely to occur later in the course of the disease as they enter into teenage years, but, these rarely constitute the initial presentation. An international multicenter stroke database showed that 90% of MMD patients initially presented with ischemic stroke, 7.5% with a transient ischemic attack, and 2.5% with hemorrhagic stroke [20]. Initial hemorrhagic presentation is seven times less likely in pediatric patients compared to adults [21]. Pediatric patients with MMD have a statistically significant increased incidence of psycholinguistic delay with, impairment of verbal, visual integration, auditory response, and executive function when compared to age-adjusted controls [22]. Headache is a common symptom in patients with MMD and is believed to be caused by irritation of the pain-sensitive fibers in the meninges by dilating neovascularization [23]. The description of these headaches is similar in quality and character to migraine-type vascular headaches. Hyperventilation has been observed to precipitate ischemic events in the pediatric population; an example that illustrates this is periods of excessive hyperventilation during a temper tantrum while the patient is crying [24]. This is caused by stenotic cortical arteries, which are supplying blood to already under perfused regions of the brain, and are reflexively vasoconstrict due to a decrease in the partial pressure of carbon dioxide. Patients are at an increased risk of sustaining an ischemic event while hypoxic, hypotensive, hypo-carbic, or hyper-thermic. Hyperthermia increases the metabolic demand and cerebral oxygen requirements, which results in an ischemic event if the stenotic vasculature is unable to compensate. Patients with MMD are at an increased risk of developing seizure-like activity due to a number of factors that can produce cortical irritation [25]. The hyperactive cortical activity that is produced during a seizure causes increased oxygen consumption in the affected region of the brain, which, if already under-perfused, can result in ischemic events.

In MMD, there is a bimodal distribution of age groups when the disease is first diagnosed; in the pediatric population, the age of initial diagnosis peaks at age five, whereas in the adult population, it is most frequently diagnosed initially in the fourth decade of life [26]. MMD has an almost 2:1 female-to-male ratio in the adult population [27]. There is a higher incidence of MMD in patients who have neurofibromatosis type I, sickle cell disease, and Down's syndrome [28]. Many of these patients are diagnosed in their late teenage years, when they are almost into adulthood. MMS is more commonly diagnosed in the adult population.

Imaging

The gold standard in image-based diagnosis and grading of Moyamoya is a catheter-based DCA. Suzuki and Takaku first classified the development of MMD into a six-stage system known as the Suzuki staging system (Table 1) [29]. 

**Table 1:** The Suzuki staging system The Suzuki staging system for Moyamoya is based on the severity of the angiographic appearance

Suzuki Stages	Angiographic appearance
Stage I	Narrowing of the terminal internal carotid bifurcation
Stage II	Initial development of the first Moyamoya collateral vessels at the base of the brain with dilation of the intracerebral main arteries
Stage III	The collateral Moyamoya vessels intensify, becoming more prominent, and the major trunks of the anterior circulation become severely stenotic and start to occlude
Stage IV	Posterior cerebral arteries become occluded, moyamoya vessels start to diminish, and collaterals from the external carotid arteries begin to form
Stage V	Moyamoya collateral vessels begin to completely disappear, and the extracranial collaterals become more and more prominent
Stage VI	Disappearance of the moyamoya collaterals and major named cerebral arteries; the cerebral hemispheres receive blood almost exclusively from abnormal external carotid anastomosis

The majority of the cases fall into Stage III, and an in-depth and more detailed staging system specifically for Stage III has been developed (Table 2) [30]. 

**Table 2:** Suzuki Stage III sub-staging system The majority of patients fall into Suzuki Stage III, and the table shows the critical subsets of Stage III scoring

Suzuki Stage III sub-stages	Angiographic appearance
Stage III A	Partial non-filling of the anterior cerebral arteries and the middle cerebral arteries
Stage III B	Partial preservation of the anterior cerebral arteries and the middle cerebral arteries
Stage III C	Complete lack of the anterior cerebral arteries and the middle cerebral arteries

Advancing angiographic stages often correlate with a deteriorating clinical progression. Pediatric populations more commonly progress through the stages while adults have a more stable course and remain in a single angiographic stage. MRI is optimal to visualize regions of the brain that have sustained acute ischemia or infarction. The appearance of a hyper-intense linear high signal sulcal pattern on the Fluid Attenuation Inversion Recovery (FLAIR) sequence is known as the “ivy sign” of MMD [31]. MRI imaging on patients with MMD shows significant flow voids in the bilateral collateral vessels to the basal ganglia and thalami, which are termed the sign of “termite nest.” This is considered virtually diagnostic for MMD [32]. The DCA is critical for selecting the branch of the ECA that will be most suitable for bypass. There are certain situations where a hypoplastic superficial temporal artery (STA) would not be ideal and a robust occipital artery (OA) would be more suitable.

The use of perfusion-weighted imaging (PWI) and CT perfusion has been instrumental in both pre and postoperative evaluation of patients with MMD. In the preoperative period, a CT perfusion scan displays regions of the salvageable ischemic brain, which contain an arterial territory that could be amenable to reperfusion with either a direct or indirect revascularization method. In cases of direct revascularization, the effectiveness of the surgery can be assessed based on the characteristics of cerebral hemodynamics on post-op CT perfusion imaging [33]. The use of transcranial Doppler (TCD) can be employed to evaluate the mean arterial velocity and resistance index before and after surgery for a comparison of the effectiveness of revascularization [34]. Serial post-operative TCDs can be used to demonstrate improved blood flow, which indicates the success of an indirect revascularization surgery, resulting in improved cerebral hemodynamics. Intra-operative Doppler ultrasound can be used to locate the STA during superficial scalp dissection and can also be used to ensure there is adequate flow through the bypass graft. Intra-operative indocyanine green fluorescent angiography can also be used intraoperatively to ensure there is adequate flow through the bypassed graft.

Treatment

Currently, there is no medical treatment proven to halt the natural progression of MMD. All medical therapies are aimed at preventing secondary complications of the disease process, such as the use of antiplatelet agents to reduce the incidence of thrombus formation as cerebral vessels progressively become occluded. Endovascular interventional stent placement has failed to prevent future ischemic events and does not halt the progression of MMD [35]. Patients with MMD are more prone to forming aneurysms than patients in the general population who are commonly treated with endovascular therapy [36]. Direct or indirect surgical revascularization is the most effective treatment option for MMD. Surgical revascularization is designed to prevent stroke and restore adequate cerebral blood flow to under-perfused regions. If a patient has bilateral disease, it is critical to address the most symptomatic side first; however, it is not uncommon to perform bilateral operations in a single surgery.

Direct Techniques

The ECA-to-ICA bypass is a surgical procedure in which the distal branches of the ECA such as the STA or OA are bypassed and directly anastomosed to distal branches of the ICA such as the M4 segment of the MCA. This re-establishes blood flow to an under-perfused region of brain parenchyma. The STA-MCA bypass, a common variant of the procedure, creates a defect in the skull to allow direct surgical anastomoses between the distal MCA (usually an M4 segment) and the STA. If the STA is not a viable option, such as when this vessel is damaged from a prior craniotomy or is congenitally hypoplastic, either the OA or middle meningeal artery (MMA) can be used to create an anastomosis with the M4 segment. The EVA distal branch such as the STA is carefully identified, preserved, and mobilized during scalp dissection. A craniotomy is performed over the desired region of the anastomosis to resupply the preferred territory of hypo-perfused brain parenchyma. A durotomy is performed and the subarachnoid space is opened to find an appropriately sized recipient M4 segment branch. The vessels are typically temporarily clamped and an incision is made in both vessels for anastomosis. There are multiple individual techniques for anastomosis that can be effective. However, it is important to dissect the adventitia off the donor vessel as this is a thrombogenic structure that will promote clot formation if it comes into contact with the lumen. Any direct bypass must suture the endothelium directly to the endothelium as the other layers in the vessel wall are highly thrombogenic. The patency of the anastomoses can be assessed intraoperatively by both a micro-Doppler or with indocyanine green. Once the patency of the graft is determined to be successful, the closure can commence. This is a highly meticulous microsurgical, technically demanding surgery that typically requires a highly skilled neurosurgeon with extensive training in cerebrovascular neurosurgery. The postoperative success of this procedure on serial angiograms ranges from 87 to 100% in various publications [37]. Mortality can range from 0.6% to 4.4% depending on how advanced the patient’s disease state is and the preoperative Suzuki grade. The incident rate of occurrence of an ischemic event related to surgery is 2.7%, transient ischemic attack (TIA) is 7.3%, seizure activity is 5.4%, and hemorrhage is 4% with a wound infection rate of less than 1% [38]. The advantage of this direct procedure is that it reestablishes blood flow immediately once the anastomosis is created.

Indirect Techniques

All indirect techniques are dependent on the physiologic process of angiogenesis as this complex biochemical process occurs in response to wound healing and allows connections to form between adjacent damaged vessels. An extensive number of angiogenic signaling molecules are upregulated in MMD, such as vascular endothelial growth factor (VEGF), fibroblast growth factor (FGF), transforming growth factor-beta (TGF-β), angiopoietin-1 (Ang1), and neuropilin-1 (NRP-1), which play a key role in collateral neovascularization [39]. Chronic increases in circulating angiogenic biochemical signaling molecules can be advantageous in indirect cerebral vasculature bypass procedures as a new anastomosis is created between external and internal circulations. The disadvantage related to indirect bypass methods is that it takes time for hemodynamically significant revascularization through angiogenesis to take place.

Encephaloduroarteriosynangiosis (EDAS) is a surgical procedure where the STA is placed into direct contact with the superficial aspect of the cerebrum without any direct anastomosis. After the craniotomy using one superior and inferior burr hole, the dura is opened, the arachnoid dissected and the pia matter opened, and the adventitial layer of the STA is dissected to remove any anatomical barrier that would prevent angiogenesis. To maintain the integrity of the vessel, the proximal portion of the STA descends into the inferior burr hole and remerges above the superior burr hole of the bone flap. Over time, micro blood vessels form an anastomosis between the STA and the distal branches of the MCA. The progressive growth of this anastomotic connection can be confirmed by serial cerebral angiograms. Eventually, flow through the STA becomes dominant and branches penetrate into the brain parenchyma, increasing hemodynamic flow. Compared to initial angiograms, serial angiograms should demonstrate a larger area of intracranial flow through the ECA. Studies have demonstrated a significant reduction in reported ischemic events from 1.7 per patient to 0.4 after EDAS, over a 38.2 month period [40]. It is very important to minimize the use of bipolar cautery with both the dura and perivascular tissue, as the microvessels in these structures are critical for the formation of collaterals in indirect techniques. 

Omental-cranial transposition is an older indirect technique that involves passing an omental flap through a subcutaneous tract superiorly through the neck. The flap is sutured into the transected dural margins and, over time, angiogenesis occurs creating an anastomosis between the rich vasculature of the omentum and distal M4 cortical branches of the MCA. The omentum is very rich in VEGF and the tissue is a strong promoter of angiogenesis [41]. However, the technique is not as widely used as omental grafts can irritate the cortex and cause an increased frequency of seizures and is often used after patients have failed prior revascularizations [42]. While the irritation is not believed to be caused by the omental vessels contacting the cerebral cortex, it is more so due to the fat, muscle, and connective tissue that is also in contact with the cortical surface. Additionally, the procedure is more invasive and involves dissections throughout layers of the abdomen, creating a tract through the chest, neck, and head, thereby causing an increased potential for complications. Some proponents disapprove of this procedure due to the higher amount of blood loss compared to more recent procedures; however, newer techniques utilizing laparoscopic techniques have been employed and they do not require a full laparotomy [43].

Encephalo-myo-synangiosis (EMS) is an indirect revascularization technique where the highly vascularized temporalis muscle is brought into direct contact with the surface of the brain. The temporalis is sutured directly into the transected edges of the dura mater. The temporalis muscle receives blood supply not only from the superficial deep temporal artery but also from deep accessory temporal arteries beneath the muscle from proximal branches of the internal maxillary artery (IMA). It is less technically demanding when compared to a direct STA-to-MCA bypass procedure. Selective external carotid angiograms performed at three months postoperatively have demonstrated extensive filling of cortical branches of the MCA from both superficial temporal and deep temporal arteries, displaying the success of the EMS [44].

Encephaloduroarteriomyosynangiosis (EDAMS) is an indirect technique that is essentially a hybrid of the EDAS and EMS surgical procedures. A vessel of the distal MCA such as the STA or OA is laid directly onto the surface of the arachnoid dissected brain's parenchyma in close approximation to distal M4 MCA segments to facilitate angiogenesis. Simultaneously, on either side of the ECA arterial indirect graft, vascular rich muscle is placed in close approximation with distal M4 segment MCA arteries, further facilitating angiogenesis.

The advantages of indirect techniques may be vast; however, it may require months to years for full revascularization. In an advanced and progressing disease process, direct techniques may be more beneficial as they provide an immediate vascular supply to under-perfused cortical areas. Studies comparing the superiority of direct versus indirect bypass show that indirect methods offer a more favorable clinical course when attempted at an earlier stage of MMD. Patients who receive indirect revascularization surgery early in the disease process have a lower overall incidence of stroke compared to those who receive conservative management with observation and medical therapy [45]. In asymptomatic patients without ischemic or hemorrhagic events, early surgical intervention has been consistently shown to have improved clinical outcomes with the prevention of future symptoms and ischemic or hemorrhagic events [46]. 

For adults with an initial presentation of an ischemic event, it appears that direct bypass is more effective in the prevention of future ischemic strokes than indirect bypass [47]. While direct bypass and combined bypass methods have demonstrated superiority in preventing future ischemic events compared to indirect bypass, neither had a statistically significant difference in the prevention of future hemorrhagic events. Patients with late-stage and advanced disease who receive direct bypass after the onset of extensive neurologic injuries did not show a reduction in ischemic stroke likelihood postoperatively compared to those who received conservative management. Early intervention when the disease process is first discovered is most likely to prevent future neurologic decline. The postoperative use of aspirin did not show a statistically significant decrease in the risk of developing postoperative stroke or increasing the patency rate of the bypass graft. However, it did not increase the risk of postoperative hemorrhage. The use of cigarettes or other tobacco-containing inhalants can be highly detrimental to any revascularization procedure and is a contraindication to proceeding with a cerebral bypass.

It is important to note that the patient is at high risk for hemorrhage postoperatively. Increased propensity for hemorrhage may be caused by a variety of different factors including reperfusion hyperemia from increased cerebral blood flow to the previously under-perfused region of the brain. It is common practice to have hyperdynamic therapy as immediate postoperative therapy to ensure the bypass graft remains patent with intravenous fluids running at 1.5 times the maintenance rate.

## Conclusions

MMD is a chronic and progressive disease without effective medical or endovascular management options; however, surgical intervention may halt the disease. Surgical treatment should strongly be considered for symptomatic patients to improve hemodynamic flow to physiologically under-perfused areas in the ICA, proximal MCA, and proximal ACA territory of brain parenchyma. In pediatric patients, early diagnosis and surgical intervention are necessary to prevent irreversible cerebrovascular infarction. Direct and indirect bypass procedures that anastomose extra-cranial to intracranial arteries have proven to be effective treatments for the prevention of ischemic and hemorrhagic strokes, regardless of age. It is critical to intervene early in the disease process before the patient becomes progressively disabled from repeated ischemic and hemorrhagic events, which means to intervene in lower Suzuki angiographic stages. Therefore, it is key that studies on MMD continually expand on available management options in order to reduce the detrimental neurologic events frequently observed in this population. When treating a patient with MMD, it is imperative to intervene early to preserve function and prevent a progressive decline. Future studies that are yet to be published are currently investigating the root underlying pathophysiological cause of MMD and may find potentially effective medical or endovascular treatments. However, currently, the only effective treatment modality is early surgical revascularization.
